# Genomic structural equation modeling reveals novel genetic loci and pathways in panvascular disease

**DOI:** 10.1097/MD.0000000000050194

**Published:** 2026-08-21

**Authors:** Lulu Gao, Baihan Jin, Kun Mei, Xiaoying Zhang

**Affiliations:** a Department of Anesthesiology, The Third Affiliated Hospital of Soochow University, Changzhou, China; b Department of Gastroenterology, No.971 Hospital of the People’s Liberation Army Navy, Qingdao, China; c Department of Ultrasound, Nanjing Hospital of Chinese Medicine Affiliated to Nanjing University of Chinese Medicine, Nanjing, China; d Department of Cardiothoracic Surgery, The Third Affiliated Hospital of Soochow University, Changzhou, Jiangsu, China.

**Keywords:** Genome-Wide Association Study, Genomic Structural Equation Modeling, Panvascular Disease, Polygenic Risk Scores, Transcriptome-Wide Association Study

## Abstract

The genetic architecture underlying panvascular disease-related traits remains poorly understood. By leveraging genomic structural equation modeling (Genomic SEM) and a variety of post-GWAS methods, we estimated causal single-nucleotide polymorphisms (SNPs) that are independent of panvascular disease variants and identified a total of 2799 genome-wide significant loci. We applied multiple transcriptome-wide association methods to identify highly correlated susceptibility gene signals and associated elements with panvascular disease from tissues, cells, and genomic elements. Subsequently, we assessed the genetic correlations across phenotypes to evaluate panvascular disease-related susceptibility factors. Additionally, polygenic scores based on summary data have been utilized to analyze evidence of panvascular disease risk across different chromosomes. Our study, through GWAS of a phenotype not directly measured, presents for the first time a comprehensive genetic landscape of panvascular disease.

## Graphical abstract

**Figure:**
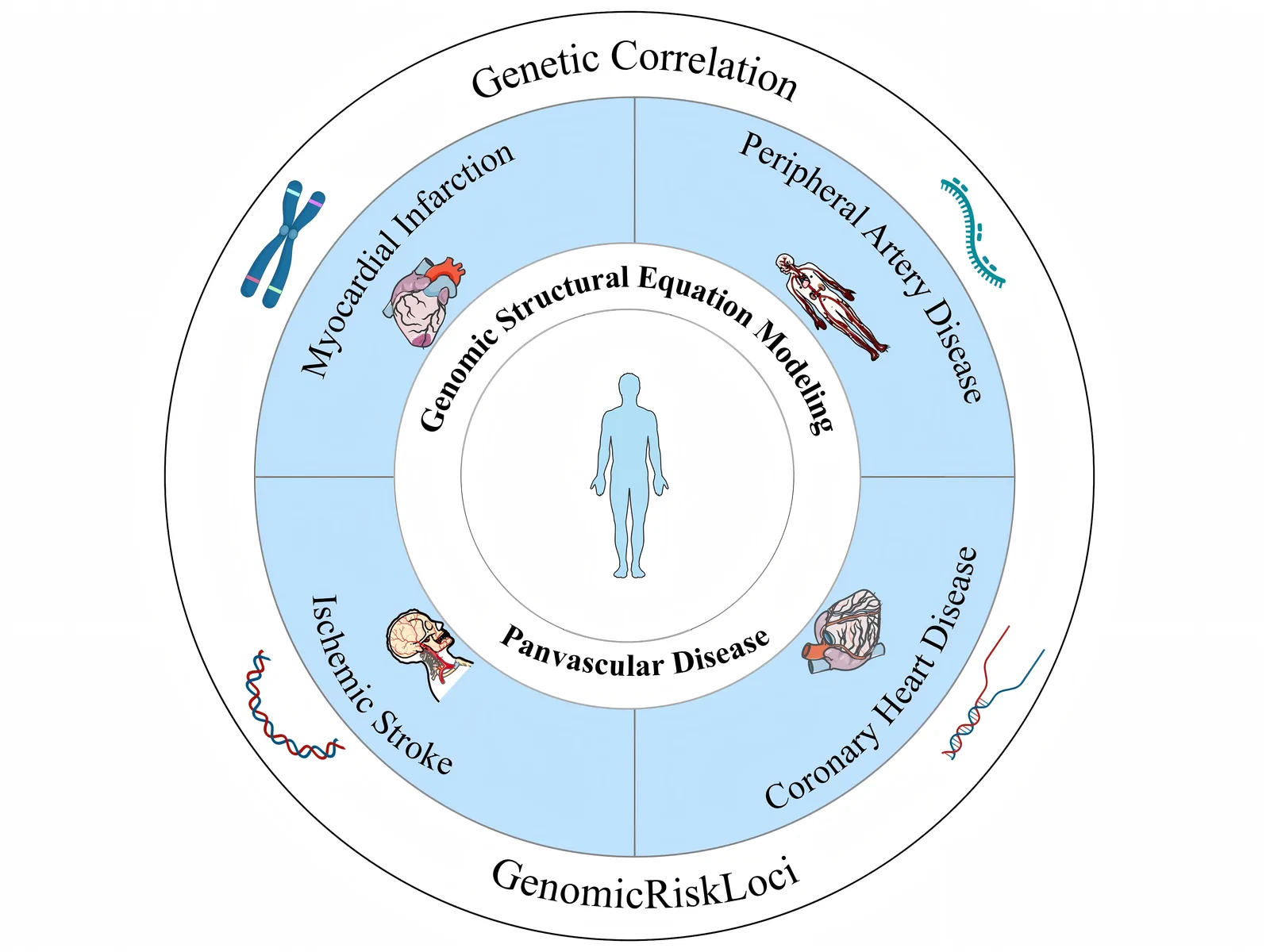

## 1. Introduction

Panvascular disease (PVD) refers to a series of vascular diseases that share atherosclerosis as a common pathological feature, primarily affecting vital organs such as the heart, brain, kidneys, and limbs.^[1]^ PVD is not only a complex and tightly interconnected mechanism but also an intricate and irreversible biological process, characterized by the deterioration of physiological functions and a decline in cellular regeneration capabilities, which are profoundly influenced by genetic, environmental, and lifestyle factors.^[2–4]^

With the intensification of global aging, the rapidly increasing incidence of PVD is becoming a significant challenge in both medical and socioeconomic fields. Despite important advancements in PVD in recent years, our understanding of its specific genetics and biology remains limited. Research indicates that atherosclerosis is a crucial driving force behind PVD, but these findings are still insufficient to fully explain the differences in the progression of PVD and disease susceptibility among individuals.^[5,6]^ To address these challenges, this study aims to explore potential molecular mechanisms and expand the connections with various potential diseases by integrating a variety of genetic analysis tools and tools for exploring strong correlations. Specifically, we focus on multiple genomic loci and chromosomal regions associated with PVD to reveal the potential for identifying therapeutic targets for PVD. This study not only expands our understanding of PVD but also provides theoretical and practical support for interventions in vascular diseases.

The latent mechanisms of PVD currently lack precise measurement. To address this, we conducted a GWAS utilizing Genomic Structural Equation Modeling (Genomic-SEM) on published summary statistics of PVD-related diseases and biomarkers. Through these summary statistics, we obtained the associations of these SNPs with the latent PVD phenotype, thereby forming a GWAS study of a latent PVD phenotype that has never been directly measured. We further drew on the comprehensive analysis methods from systems biology, defining the genetic variations in the PVD structural equation not explained by known biomarkers as latent relevant genetic markers and conducted various GWAS-related studies on them. Although this method is not perfect in reflecting the true relationship between PVD-related pathways and multifactorial interactions, as the PVD structural equation is a complex process driven by genetic, environmental, and stochastic factors, this analysis excludes confounding influences based on PVD biomarkers, thus enabling the analysis of data that would otherwise be difficult to study. Finally, we conducted tens of thousands of correlation analyses to construct a map of PVD influencing factors, with the aim of allowing non-biostatisticians to directly apply the relevant influencing factor maps to designate potential prevention and intervention measures for patients. Our study aims to pioneer a simple pathway from genomic statistics to basic research and the formulation of clinical measures.

## 2. Materials and methods

### 2.1. Data sources for single-input GWAS

The single-input GWAS data for our PVD structural equation GWAS were derived from four related GWASs, including myocardial infarction (ukb-b-15829, n = 462,933), ischemic stroke (ieu-a-1108, n = 29,633), peripheral artery disease (ukb-d-I9_PAD, n = 361,194), and coronary heart disease (ukb-d-I9_CHD, n = 361,194). All input GWASs had ethical permissions from their respective institutional review boards, and all participants provided informed consent with rigorous quality control.

### 2.2. Quality control for single-input GWAS

#### 2.2.1. Exclusion of low-quality samples

Samples with a missing rate exceeding 5% were excluded.

#### 2.2.2. MHC region

The Major histocompatibility complex (MHC) region (located on chromosome 6 between approximately 25,000,000 and 35,000,000 bp) was treated separately due to its genetic diversity and complex structure, particularly the polymorphisms of immune-related genes.

#### 2.2.3. Preparation of summary statistics for PVD structural equation

We used the default parameters for preparing the summary statistics. Please refer to **Supplementary Material 1**, Supplemental Digital Content 1 for more details.

### 2.3. Sample overlap in single-input GWAS

The single-input GWASs included in our analysis were sourced from different genomic databases with distinct participant pools. This ensured that our GWAS analysis accounted for potential sample overlap between cohorts, thereby enhancing the accuracy and generalizability of the results. We also considered the statistical implications of potential sample overlap.

### 2.4. Genomic structural equation modeling

We conducted a multivariate GWAS of myocardial infarction, ischemic stroke, peripheral artery disease, and coronary heart disease using Genomic SEM as implemented in the GenomicSEM^[7]^ R package (v.0.0.5). This recently developed method is unbiased by sample overlap or imbalanced sample sizes and can identify variants influencing only a subset of traits, in addition to those with broad cross-trait effects. The analysis proceeded in 2 stages; please see **Supplementary Material 1**, Supplemental Digital Content 1 for full details.

### 2.5. Genomic SEM SNP heterogeneity

To evaluate whether the PVD SNP associations are appropriately modeled within a multivariate SEM framework, we calculated SNP heterogeneity statistics. We assessed QSNP heterogeneity with a significance threshold of *P* < .05. Please refer to **Supplementary Material 1**, Supplemental Digital Content 1 for more details.

### 2.6. Multilevel assessment of genomic structural equation model

We performed a multilevel strategy for the constructed genomic structural equation model, which included adjusting the p-values for correlation tests (*P* < 5 × 10^−16^, *P* < 5 × 10^−12^) to identify novel SNP loci at different levels. We also used a two-step LD Score regression approach with the following steps. Please refer to **Supplementary Material 1**, Supplemental Digital Content 1 for more details.

### 2.7. Defining genomic loci and identifying novel variants

We used FUMA^[8]^ (Functional Mapping and Annotation) to identify genomic loci and lead SNPs associated with PVD, which have low linkage disequilibrium (LD) with other SNPs (<0.1) and are genome-wide significant (*P* < 5 × 10^*−*8^). Please refer to **Supplementary Material 1**, Supplemental Digital Content 1 for more details. Furthermore, we developed an interesting method (named GWAS subtraction method) by comparing the lead loci identified by genomic SEM with those reaching genome-wide significance in single-input GWAS, which can effectively discover more valuable novel loci.

### 2.8. Fine mapping

To identify the most plausible causal variants associated with PVD, we used SuSIE and FINEMAP,^[9]^ using the R package echolocatoR (v.2.0.3), we implemented an analysis with a probability threshold of 0.95 to define credible sets of potentially causal variants. The detailed steps are as follows:

Identifying Causal Variants with SuSIE and FINEMAP: SuSIE (Sum of Single Effects) and FINEMAP are tools for fine-mapping analysis aimed at determining the most likely causal variants associated with a phenotype. Please refer to **Supplementary Material 1**, Supplemental Digital Content 1 for more details.

Credible Sets: We set a probability threshold of 0.95, and if a variant’s posterior probability exceeded this threshold, it was considered a potential causal variant.

Consensus SNPs and Probability Sets: echolocatoR defines a ‘consensus SNP’ as a variant that appears in both SuSIE and FINEMAP results. Please refer to **Supplementary Material 1**, Supplemental Digital Content 1 for more details.

### 2.9. Transcriptome-wide association study (TWAS)

After identifying potential causal variants, we performed a transcriptome-wide association study (TWAS) to prioritize genes linked to peripheral vascular disease (PVD) based on the correlation between gene expression and phenotype. We utilized the FUSION method for TWAS, leveraging 37,920 precomputed eQTL features from GTEx v.8 (genetissue pairs) to assess expression relationships across various genes and tissues. For more details, please see **Supplementary Material 1**, Supplemental Digital Content 1.

### 2.10. Gene set and disease ontology enrichment analysis

We performed gene enrichment and pathway set analysis using MAGMA and FUMA (GESA) data to investigate the potential relationships of PVD with Mendelian disease genes and associated pathways.

### 2.11. Cell-type annotation analysis

To identify etiological cell types associated with PVD, we integrated single-cell RNA sequencing data using CELLECT.^[10]^ We used the Tabula Muris dataset, which contains transcriptomic data from 100,000 cells and 20 organs and tissues of Mus musculus.^[11]^ We preprocessed and normalized the data using CELLEX, calculating expression specificity likelihood scores for each gene. We then performed cell-type-specific analysis using the ldsc software, classified cell types, and applied a false discovery rate (FDR) threshold of 0.05.

### 2.12. Genetic contribution of genomic regions

The LDSC tool was used to calculate partitioned heritability, which allocates the genetic information of a phenotype to different genomic regions (such as genes, enhancers, and repressors) to assess their contributions to phenotypic heritability. Specifically, LDSC uses weighted LD matrices, genotype frequency files, and summary statistics for calculation, ultimately estimating the genetic contribution of each region.

### 2.13. Cross-phenotype genetic correlations

To identify the associations between the constructed unmeasured PVD GWAS analysis data and previously studied diseases and biomarkers, we performed large-scale association analyses. We used the iCPAGdb database for cross-phenotype analysis to study the genetic correlations between PVD GWAS and other traits. We implemented Mendelian randomization (MR) analysis using the MendelianRandomization R package v.0.7.0, with inverse variance weighting (IVW) as the primary method for correlation assessment (*P* < .05).

### 2.14. Polygenic risk score construction based on summary data

We calculated polygenic risk scores (PRS) based on genome-wide summary statistics and assessed the genetic contribution of different chromosomal regions to the disease. Specifically, We computed polygenic risk scores (PRS) using PRS-CS, a Bayesian approach that applies continuous shrinkage priors to estimate SNP effect sizes from GWAS summary statistics, with linkage disequilibrium (LD) estimates derived from an external reference panel.

## 3. Results

### 3.1. Structural equation model (SEM) statistics construction

**Figure 1.**
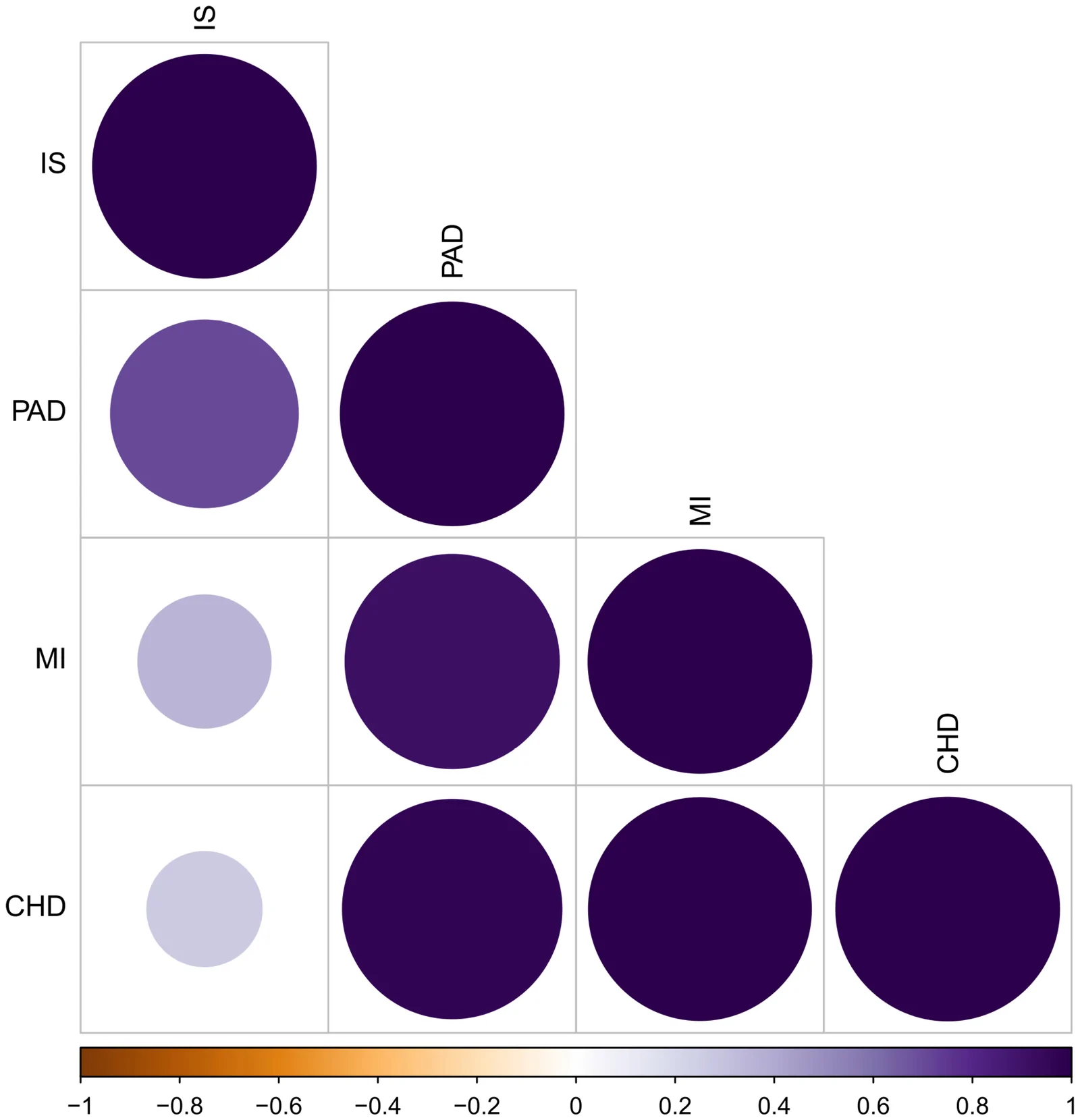
LD-Score assessment of genetic correlations for each variable.

The genetic contributions of the four single-input GWASs (MI, IS, PAD, CHD) were estimated using LD-Score regression analysis, with heritability estimates as follows: MI: H2, Z = 11.8; IS: H2, Z = 4.5; PAD: H2, Z = 2.01; CHD: H2, Z = 10.4 (detailed single-factor genetic parameters are provided in Table S1, Supplemental Digital Content 2 and Figure 1). Prior to modeling, we conducted structural equation model analysis. The common factor model fit for the genetic covariance matrix and empirical covariance matrix of the four input GWASs was good (Comparative Fit Index (CFI) = 0.998876178300326, Chi-square to degrees of freedom ratio (X^2^/df) = 1.332852449) (detailed model stability assessment and parameters of the latent factor (F1) and single-variable structural equation model are provided in Table S2, Supplemental Digital Content 3), indicating evidence for a shared genetic factor underlying PVD.

### 3.2. Hierarchical assessment of genomic structural equation model GWAS

By extending the Structural Equation Model (SEM) to incorporate individual variants, we generated an indirect measurement GWAS, estimating the associations of 2200,872 single nucleotide polymorphisms (SNPs) with PVD. We identified 164 lead SNPs (*P* < 5 × 10^−16^) across 340 genomic loci (*P* < 5 × 10^−12^) (Table S3, Supplemental Digital Content 4). Among these 164 SNPs, 57 were novel compared to the loci identified in the four single-input GWASs used to construct PVD, highlighting the enhanced power of genomic SEM. The novel PVD lead SNPs were generally enriched for traits such as myocardial infarction, aspirin usage, heart failure, antithrombotic drug usage, and peripheral artery disease (Table S4, Supplemental Digital Content 5). Several of these SNPs have been previously highlighted in the literature (rs9349379, rs660240, and rs602633) as being associated with PVD traits, while others (rs1333037, rs2184061, and rs3218020) were not directly linked to specific PVD GWAS phenotypes but were significantly associated with indirect factors that may influence PVD traits (such as abnormal blood perfusion and impaired vascular regulation). This study, by introducing genomic SEM, revealed SNPs associated with PVD under different correlation scenarios, and these newly discovered SNPs are often related to important factors influencing PVD.

### 3.3. Genomic structural equation model based on LD score regression genomic control assessment

A total of 1207,387 SNPs were removed through parameter control in the method, and 993,483 SNPs were retained with regression coefficients. The average chi-square value for all SNPs was Mean Chi^2^ = 2.032, with a genomic control Lambda GC = 1.895. The maximum chi-square value was Max Chi^2^ = 171.073, and the genome-wide significance level was 1562. The heterogeneity test passed (*P* > .05). The total observed scale heritability (h^2^) was -8.0559e-05 (5.1888e-06), and the intercept in the regression model was 2.6887 with a standard error of 0.0229. These estimates directly indicate that the inflation observed in the structural equation of interest is due to polygenic heritability signals rather than population stratification bias and pleiotropic effects.

### 3.4. Evaluation of PVD structural equation model based on FUMA software

Using the FUMA software, we identified a total of 1825 risk loci (Table S5, Supplemental Digital Content 6, Figure 2) and 2322 potential PVD-related genes through genome-wide significance control (sig.thres = 5e-8, FDR < 0.05, Table S6, Supplemental Digital Content 7, Figures 3). A total of 2638 lead SNPs were annotated, with the majority located in intergenic regions (Table S7, Supplemental Digital Content 8). The GWAS subtraction method identified 9 SNPs that were only studied in single traits (rs17011681, rs6511720, rs660240, rs9349379, rs3731239, rs4766578, rs6857, rs10455872, rs1537373) (Table S8, Supplemental Digital Content 9). Among these, rs17011681, rs660240, rs3731239, rs4766578, rs6857, rs10455872, and rs1537373 have been reported in multiple studies, but we found that these SNPs are not directly causative for PVD but rather potential mediating loci. rs6511720 and rs9349379 have been consistently associated with the phenotypes studied in previous literature (Table S9, Supplemental Digital Content 10).

**Figure 2.**
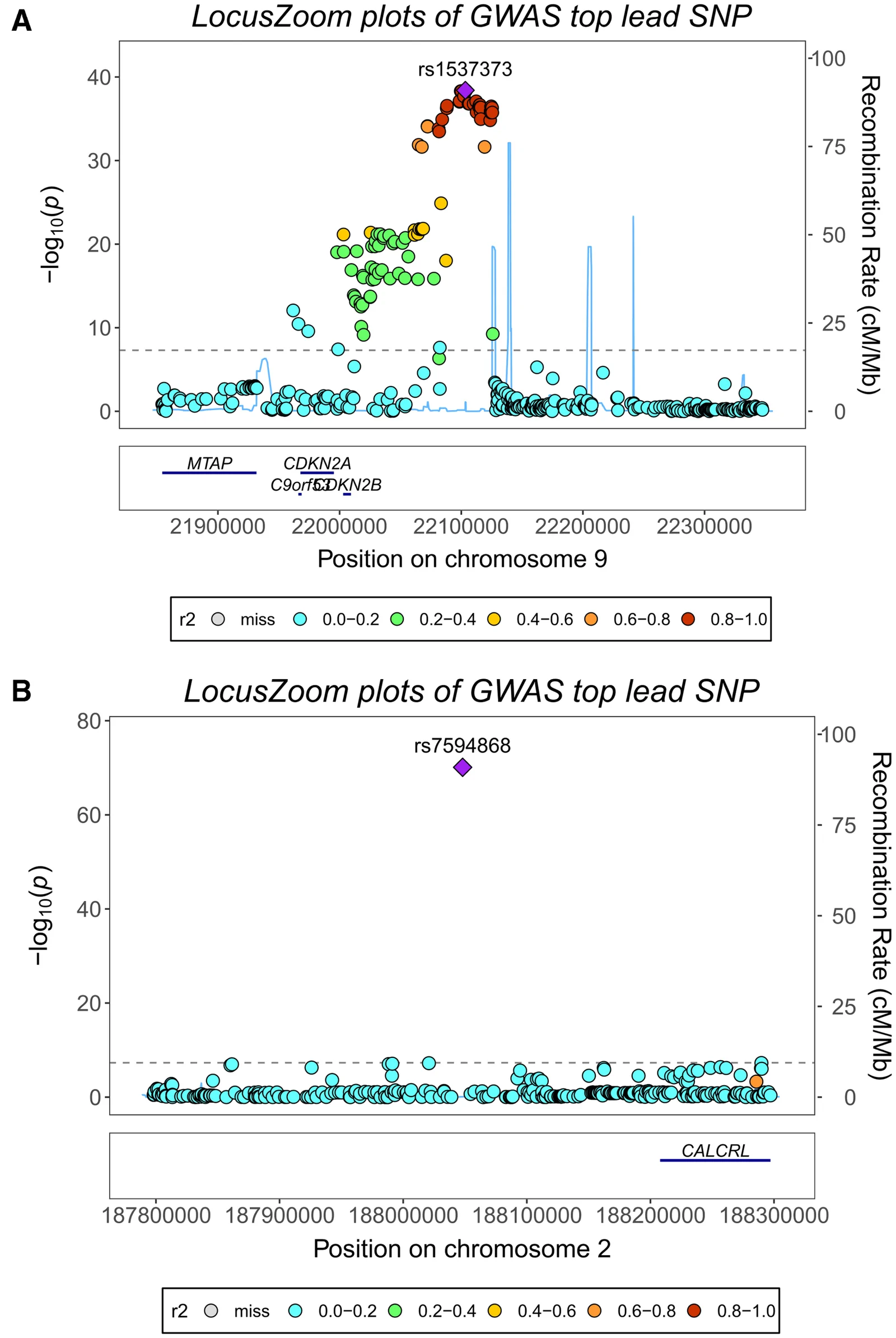
Risk loci identified in the PVD structural equation evaluated using FUMA software (we plotted the top two significant risk loci - rs1537373 (A), rs7594868 (B)).

**Figure 3.**
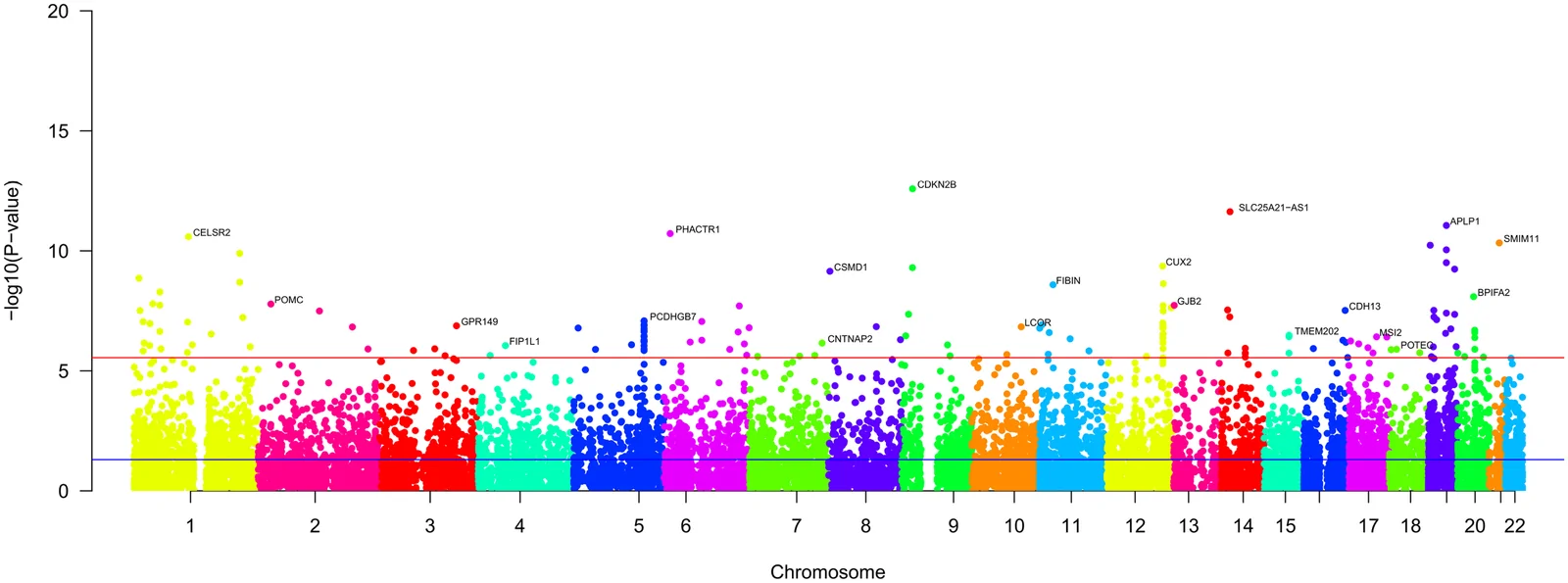
Manhattan plot identifying a total of 2322 potential PVD-related genes.

### 3.5. Fine mapping

**Figure 4.**
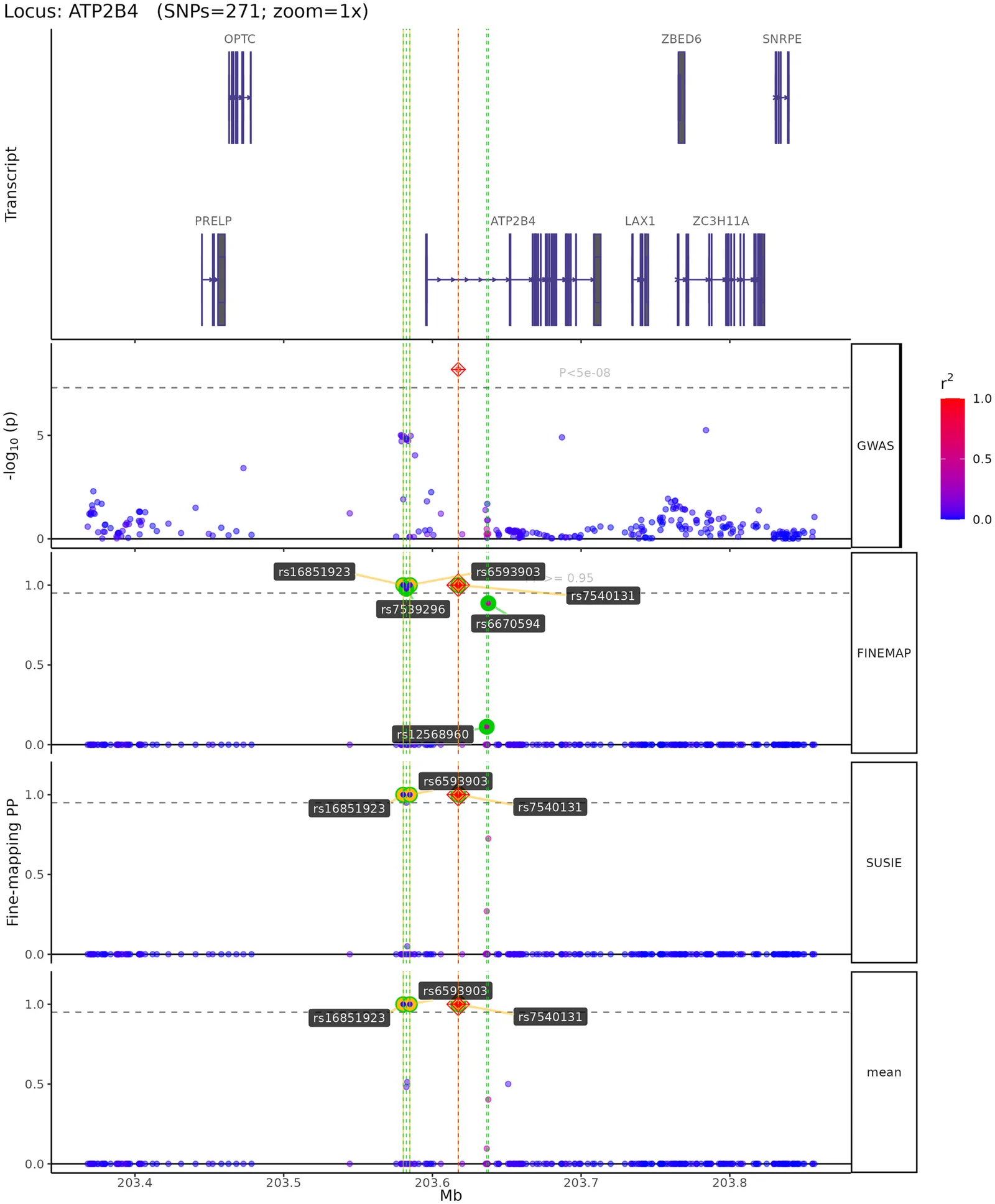
Regional plot of the ATP2B4 variant on chromosome 1.

Fine mapping analysis identified strong associations at multiple genomic loci, including: chromosome 1 (rs16851923 and rs6593903, variants in ATP2B4); chromosome 5 (rs42278, variant in CTNND2 locus); chromosome 10 (rs3750641 and rs3750644, variants in ANKRD16 locus); chromosome 17 (rs7219918, variant in SAP30BP locus); and chromosome 18 (rs15101, variant in SS18 locus) (Table S10, Supplemental Digital Content 11). Regional plots show clear peaks at these loci, with other credible set variants also showing evidence of association (Fig. 4).

### 3.6. Transcriptome-wide association study

We performed a transcriptome-wide association study (TWAS) using FUSION to identify gene-level associations with PVD. We found 77 genes surpassing the threshold for multiple comparisons (Table S11, Supplemental Digital Content 12, Figure 5). Subsequent FOCUS fine mapping analysis identified 239 genes (Table S12, Supplemental Digital Content 13, Figure 6) that may represent causal signals for PVD. To further confirm these “high-confidence” gene-level associations, we conducted colocalization tests. This included PRRT1, RPS18, AKT1, and TCF19. The TWAS Z scores for AKT1 were all >0, indicating that predicted gene expression is positively correlated with PVD, suggesting that upregulation of these genes may be associated with increased PVD. Conversely, the TWAS Z scores for PRRT1, RPS18, and TCF19 were <0, indicating that their downregulation is associated with increased PVD (Table S13, Supplemental Digital Content 14).

**Figure 5.**
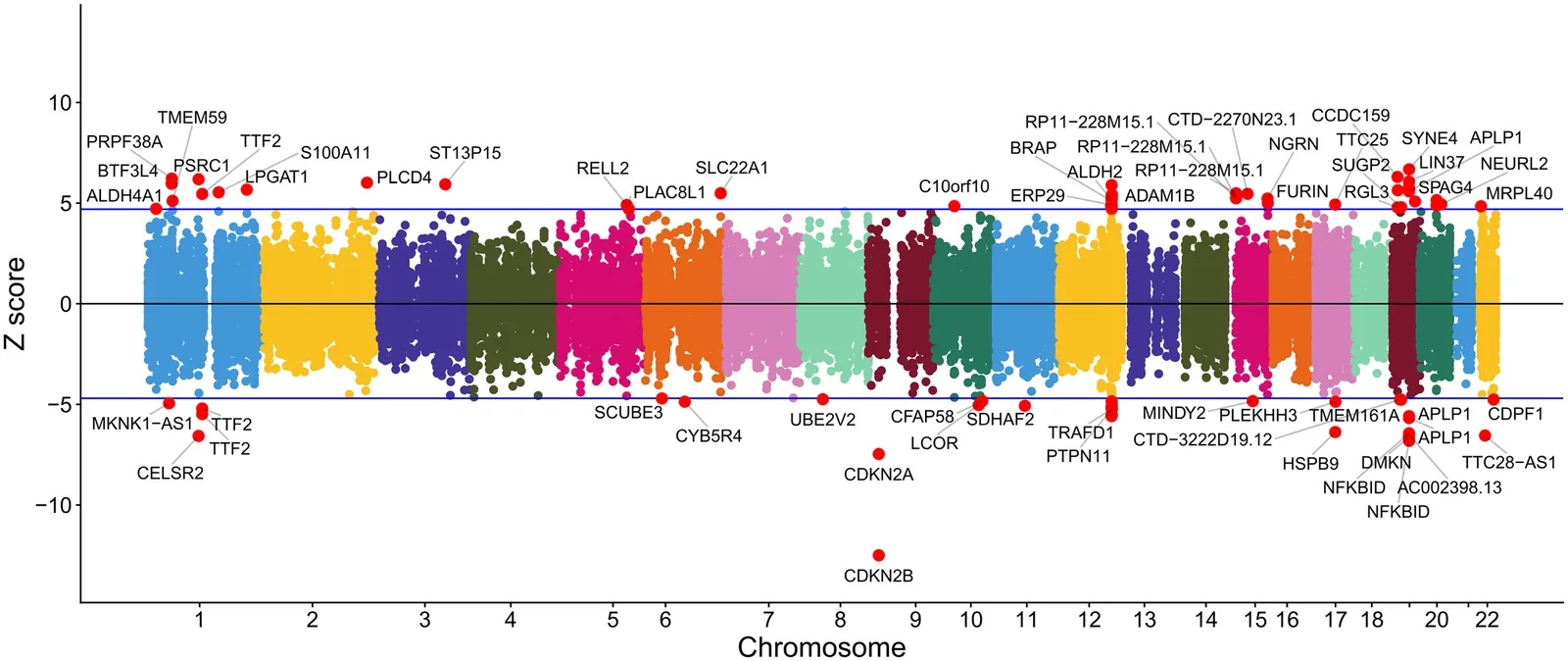
TWAS analysis identifying 77 gene-level associations with PVD traits.

**Figure 6.**
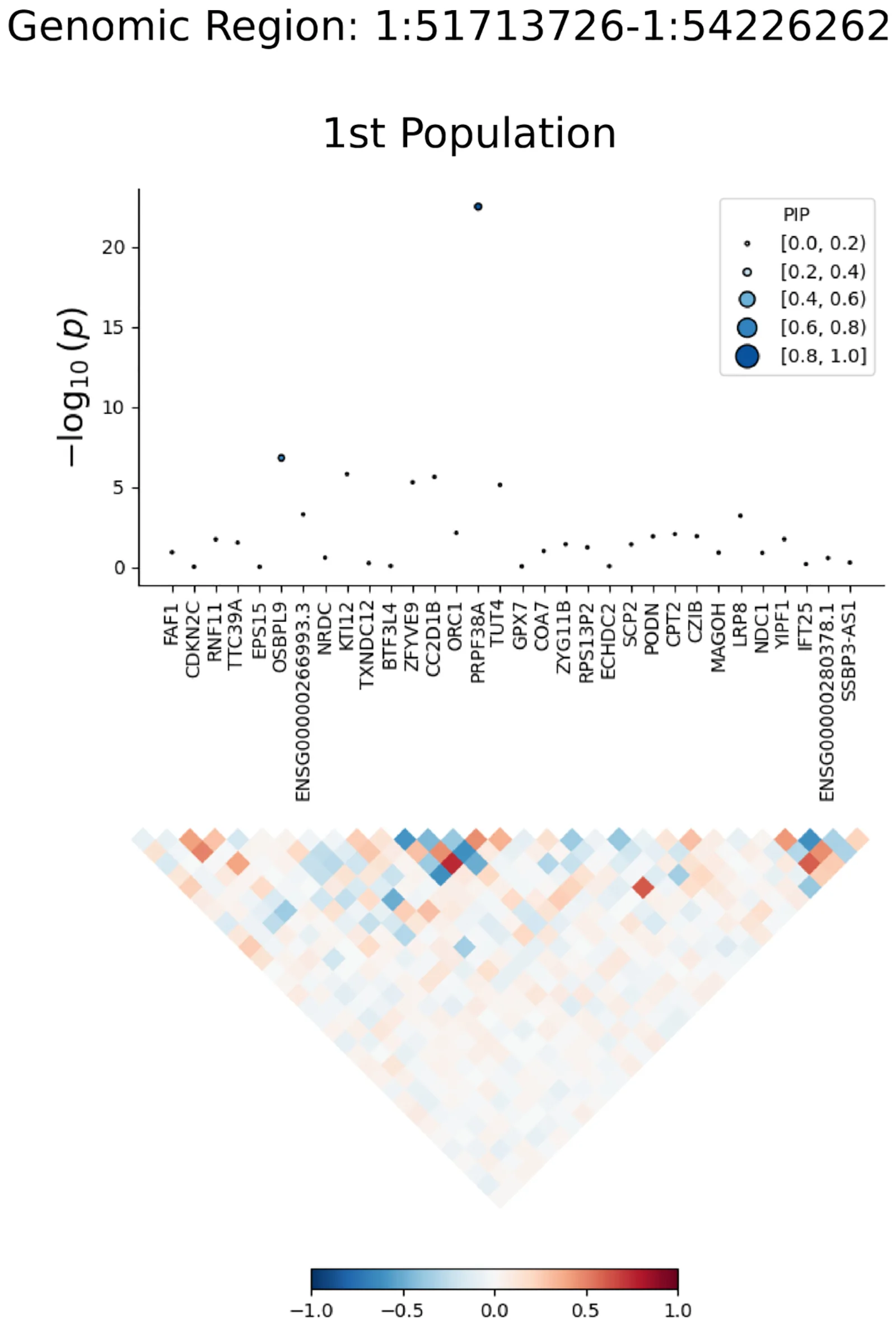
Gene-level associations from FOCUS fine-mapping (we plotted significant gene loci).

### 3.7. Pathway, cell-type, and Mendelian disease gene enrichment

Multimarker analysis of genomic annotation (MAGMA) gene mapping identified 149 genes (Table S14, Supplemental Digital Content 15), which we used for gene set analysis. These genes were enriched in GSEA terms (Table S15, Supplemental Digital Content 16), with many gene sets related to blood pressure (e.g., systolic blood pressure, mean arterial pressure) and aging. Cell-type enrichment analysis showed that 9 cell types exceeded the significance threshold after multiple comparisons correction. The most significant cell type was fibroblasts (Tables S16–19, Supplemental Digital Content 17).

### 3.8. Genomic region heritability contribution results

In the analysis of genetic region heritability contribution, it was discovered that the majority of heritability contribution sites are clustered in regulatory regions and gene-enriched sections of chromosomes. These areas are typically crucial for regulating gene expression, modifying chromatin, and binding transcription factors. Specifically, the influence of genetic variation is most prominent in promoter and enhancer regions, which may have a significant impact on trait or disease susceptibility by controlling gene expression levels. Furthermore, certain noncoding regions also exhibit significant heritability contributions, indicating their potential involvement in complex genetic processes through the regulation of gene expression or function (Table S20, Supplemental Digital Content 18).

### 3.9. Cross-phenotype analysis

Using iCPAGdb, we conducted cross-phenotype genetic correlation analysis on PVD genome-wide significant SNPs. The correlated traits were mainly concentrated in BMI and vascular-related indicators (Table S21, Supplemental Digital Content 19, Figure 7). We downloaded GWAS summary statistics for 18 traits from the GWAS Catalog for genetic correlation analysis. After multiple corrections, systolic blood pressure, BMI, pulse pressure, body fat percentage, and peripheral artery disease showed positive correlations with the constructed structural equation, while height and educational attainment showed negative correlations (Table S22, Supplemental Digital Content 20).

**Figure 7.**
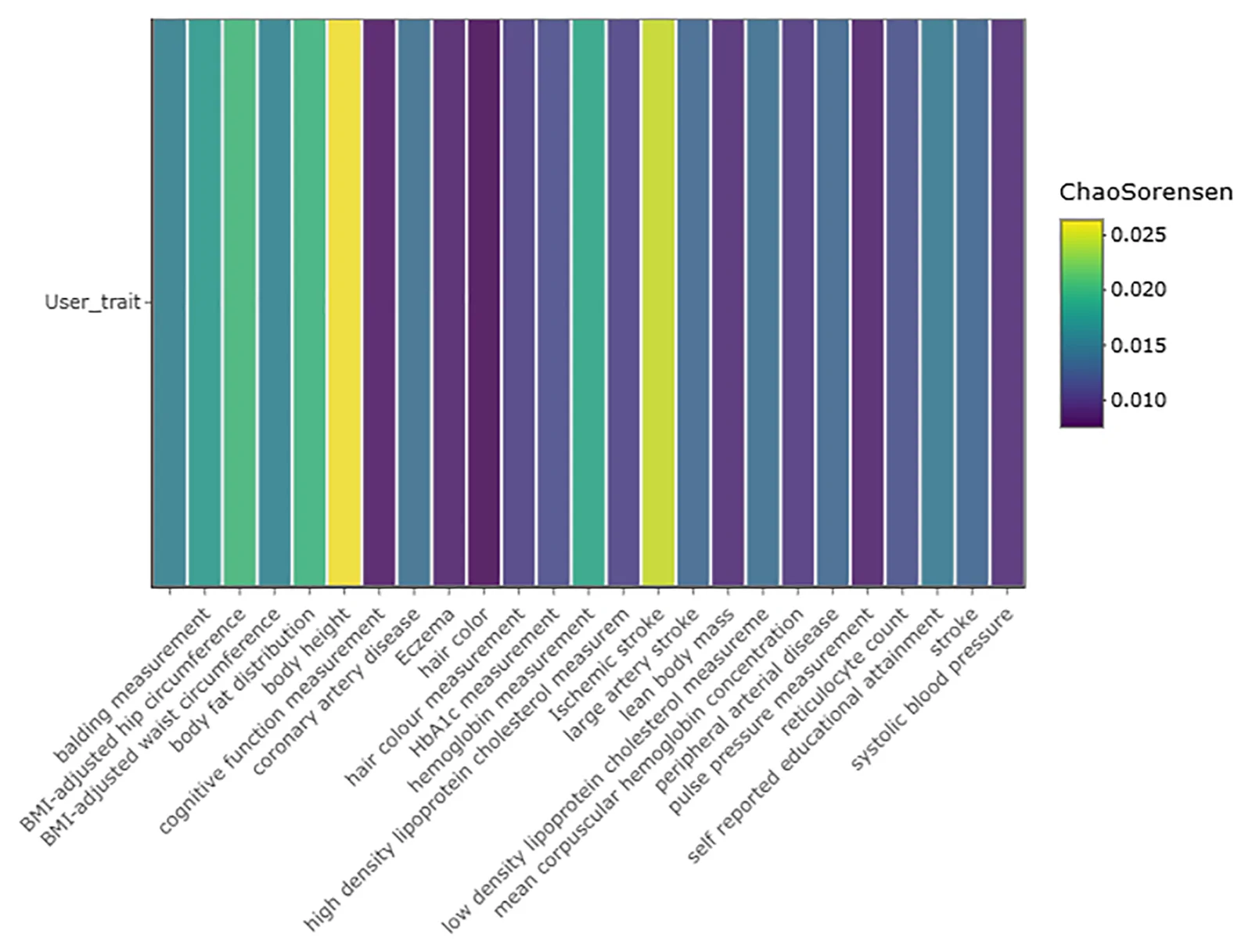
Heatmap of significant cross-phenotype genetic correlations for PVD genome-wide significant SNPs analyzed using iCPAGdb.

### 3.10. Chromosome-level results

Our analysis indicates that PRS-constructed variants are closely related to the risk of PVD, and there are significant differences in genetic contributions from different chromosomal regions to the disease. Particularly in regions such as chromosomes 3 and 9, we observed higher genetic contributions, which may contain important genes and regulatory elements affecting disease susceptibility (Table S23, Supplemental Digital Content 21).

## 4. Discussion

This study leverages Genomic Structural Equation Modeling (Genomic SEM) and a variety of post-GWAS methods to delve into the genetic architecture of PVD, identifying numerous novel genetic markers and thereby forging new pathways for comprehending the genetic underpinnings of PVD. Previous studies have predominantly focused on individual vascular diseases. In contrast, this study adopts a panvascular perspective, taking into account the shared genetic characteristics of multiple vascular diseases, which aids in gaining a more comprehensive grasp of the pathogenesis of PVD. Moreover, the design concept of conducting GWAS research on PVD phenotypes that have not been directly measured offers fresh ideas for genetic studies of similar complex diseases.

A total of 2799 genome-wide significant loci were identified in this study, with 57 of the 164 lead SNPs being novel discoveries across different *P*-value thresholds. These novel loci highlight the robust capability of the Genomic SEM in uncovering potential PVD-related genetic variations. The enrichment traits of these loci encompass a variety of factors closely associated with PVD, such as myocardial infarction, aspirin usage, and heart failure, suggesting that these genetic variations may influence the occurrence and development of PVD through multiple biological pathways. Through fine-mapping tools like SuSIE and FINEMAP, key variant sites on multiple chromosomes were identified, such as the ATP2B4 variant on chromosome 1 and the CTNND2 locus variant on chromosome 5. The discovery of these sites provides clear targets for further exploration of their biological functions. Most of these newly discovered SNPs are located in gene regulatory regions, indicating that noncoding regions may play an important role in genetic mechanisms. This is consistent with the findings of Kellis et al,^[12]^ who pointed out that variations in noncoding regions may influence the formation of complex traits by regulating gene expressionwho pointed out that variations in noncoding regions may influence the formation of complex traits by regulating gene expression.

Another important finding of this study is the identification of several novel risk factors that have not been widely reported in previous PVD or similar studies. These newly identified susceptibility factors may be associated with specific biological pathways and provide new directions for further clinical research and the development of drug target.^[13]^ For example, certain genetic variations may serve as potential drug targets, and future drug development could be designed to target these variations, thereby achieving more precise treatments.

By analyzing whole-genome data, we identified multiple risk chromosomal regions associated with the disease, many of which are located in noncoding regions. Research indicates that these regions influence a variety of biological processes, including cell proliferation, differentiation, and immune responses, by regulating the expression of nearby genes.^[14,15]^ For example, we identified several risk loci on chromosomes 3 and 9, which are associated with traits such as neurodevelopment and cardiovascular diseases and are often enriched in functional gene regulatory regions, such as enhancers and promoters. These genomic elements often have a lasting impact on specific biological processes by regulating the spatiotemporal patterns of gene expression. For complex diseases like cardiovascular disease and cancer, studies have pointed out that these genomic regulatory regions are closely related to the expression of disease-associated genes.^[16]^ We also found that in some risk chromosomal regions, changes in the expression of noncoding RNAs are closely related to disease susceptibility, indicating that these regions may not only affect gene expression but also influence disease occurrence by regulating RNA levels.

Despite providing new insights into the genetics of PVD, this study has several limitations.

The initial study cohort primarily consists of individuals of European ancestry, with limited validation in other populations. Therefore, future research should expand the sample coverage to include more diverse ethnic and regional groups, which is crucial for ensuring the broad applicability of the genetic findings. Furthermore, while our detailed mapping and transcriptomic analyses have identified multiple genetic loci associated with PVD, connecting these genes to specific biological mechanisms remains a pressing challenge. Subsequent investigations should delve deeper into how these genetic variants influence individual PVD outcomes by modulating gene expression, neurodevelopment, and metabolic pathways. Additionally, although our study underscores the significant role of genetic factors in PVD, the influence of environmental factors should not be overlooked. Future work should further explore how environmental exposures interact with genetic predispositions to affect phenotypic expression, thereby promoting in-depth research into gene-environment interactions.

## 5. Conclusion

This research provides new insights into the genetic foundation of PVD. Utilizing Genomic Structural Equation Modeling, fine-mapping, and transcriptomic analyses, we have identified numerous novel genetic loci and elucidated their impact on gene expression and their genetic connections to complex traits. Our discoveries not only advance the understanding of the genetic mechanisms of PVD, but also present new avenues for precision medicine and public health interventions. Subsequent studies will confirm these genetic markers and investigate the role of gene-environment interactions in health and educational outcomes, with the goal of enhancing healthy life expectancy and educational attainment on a global scale.

## Author contributions

**Conceptualization:** Lulu Gao.

**Funding acquisition:** Lulu Gao.

**Writing – original draft:** Lulu Gao, Baihan Jin.

**Data curation:** Baihan Jin.

**Investigation:** Baihan Jin.

**Resources:** Kun Mei.

**Supervision:** Kun Mei, Xiaoying Zhang.

**Writing – review & editing:** Kun Mei, Xiaoying Zhang.

**Visualization:** Xiaoying Zhang.










































