# Supplementary material for: Genomic structural equation modeling reveals novel genetic loci and pathways in panvascular disease
Source: Medicine (Baltimore). 2026 Aug 21;105(34):e50194. doi: 10.1097/MD.0000000000050194 (PMC13502809; doi:10.1097/MD.0000000000050194)
Supplement: Supplementary file 1 [file medi-105-e50194-s001.docx]

**Data Sources for Single-Input GWAS**

The single-input GWAS data for our PVD structural equation GWAS were derived from four related GWASs, including myocardial infarction (ukb-b-15829, n = 462,933), ischemic stroke (ieu-a-1108, n = 29,633), peripheral artery disease (ukb-d-I9_PAD, n = 361,194), and coronary heart disease (ukb-d-I9_CHD, n = 361,194). All input GWASs had ethical permissions from their respective institutional review boards, and all participants provided informed consent with rigorous quality control.

**Quality Control for Single-Input GWAS**

1) Exclusion of Low-Quality Samples: Samples with a missing rate exceeding 5% were excluded.
2) MHC Region: The Major histocompatibility complex (MHC) region (located on chromosome 6 between approximately 25,000,000 and 35,000,000 bp) was treated separately due to its genetic diversity and complex structure, particularly the polymorphisms of immune-related genes.
3) Preparation of Summary Statistics for PVD Structural Equation: We used the default parameters for preparing the summary statistics. We filtered all autosomal SNPs from the four PVD-related GWASs using the recommended default quality control filters, restricting to the 1000 Genomes Phase 3 EUR panel. SNPs with MAF < 0.01 were removed (these SNPs are prone to error due to fewer samples within the genotype cluster and higher LD score regression standard errors). SNPs with effect values estimated to be exactly zero were also removed (to avoid compromising matrix inversion necessary for genomic SEM). SNPs not matched with the reference panel and SNPs with mismatched alleles were excluded.

**Sample Overlap in Single-Input GWAS**

The single-input GWASs included in our analysis were sourced from different genomic databases with distinct participant pools. This ensured that our GWAS analysis accounted for potential sample overlap between cohorts, thereby enhancing the accuracy and generalizability of the results. We also considered the statistical implications of potential sample overlap.

**Genomic Structural Equation Modeling**

We employed Genomic SEM implemented in the GenomicSEM[7] R package (v.0.0.5) to conduct a multivariate GWAS analysis of myocardial infarction, ischemic stroke, peripheral artery disease, and coronary heart disease, investigating the broad genetic liability underlying these traits. Genomic SEM is a recently developed method that allows for the investigation of multiple potential multivariate models of the underlying architecture of the traits of interest.

Genomic SEM is not biased by sample overlap or imbalanced sample sizes. It also facilitates the identification of variants that influence only some but not all of the complex traits, and which therefore do not represent a broad cross-trait liability.

Genomic SEM proceeds in two stages. Stage 1 estimates the empirical genetic covariance matrix and corresponding sampling covariance matrix. We prepared the PVD-related GWAS summary statistics for Stage 1 and used the multivariate extension of cross-trait LD score regression to generate the empirical genetic covariance matrix between the four traits as input for the SEM common factor model. Stage 2 specifies an SEM that minimizes the hypothesized covariance matrix and the empirical covariance matrix calculated in Stage 1. Here, our primary study aim was to identify a genetic signature underlying the four PVD-related traits; we therefore tested a one-factor model. Model fit was assessed using the SRMR, model χ², Akaike information criterion, and CFI.

After applying the appropriate common factor SEM specification, individual autosomal SNP associations were incorporated into the genetic and associated sample covariance matrices to generate a multivariate genome-wide analysis (GWAS) of the shared covariance across the four PVD-related GWASs.

**Genomic SEM SNP Heterogeneity**

To evaluate whether the PVD SNP associations are appropriately modeled within a multivariate SEM framework, we calculated SNP heterogeneity statistics. The null hypothesis of the QSNP test is that the SNP associations on the single-phenotype GWASs are statistically mediated by PVD. Therefore, significant QSNP tests in PVD would suggest that the SNP impacts the single-phenotype GWASs by pathways other than the shared genetics of atherosclerosis modeled by PVD. We assessed QSNP heterogeneity with a significance threshold of *P < 0.05*.

**Multilevel Assessment of Genomic Structural Equation Model**

We performed a multilevel strategy for the constructed genomic structural equation model, which included adjusting the p-values for correlation tests (*P < 5 × 10⁻¹⁶*, *P < 5 × 10⁻¹²*) to identify novel SNP loci at different levels. We also used a two-step LD Score regression approach with the following steps: no removal of SNPs with missing values, no removal of SNPs with INFO values less than 0.9, no removal of SNPs with MAF less than 0.01, no removal of SNPs with P-values outside the valid range, no removal of non-SNP or ambiguous strand SNPs, and removal of partitions with zero variance LD scores. We used a two-step estimator with a cutoff value of 30.

**Defining Genomic Loci and Identifying Novel Variants**

We used FUMA [8] (Functional Mapping and Annotation) to identify genomic loci and lead SNPs associated with PVD, which have low linkage disequilibrium (LD) with other SNPs (<0.1) and are genome-wide significant (*P < 5 × 10⁻⁸*). First, we extracted the summary statistics for these lead PVD SNPs to assess their association strengths. We also compared the lead SNPs and loci with the original single-variable GWAS and defined loci as novel if they were >1 Mb from loci identified in the single-variable GWAS data. To determine whether any of the N lead SNPs in PVD showed evidence of pleiotropic associations, we also looked up published GWAS-significant associations (*P < 5 × 10⁻⁸*) in the GWAS Catalog. Additionally, we performed risk locus analysis on the established PVD model using the FUMA software, with a significance threshold of *P< 5 × 10⁻⁸*, and analyzed the output files with MAGMA (Multi-marker Analysis of GenoMic Annotation), a tool for post-GWAS analysis aimed at evaluating gene-phenotype associations. The significance threshold was set at FDR *P < 0.05*. Furthermore, we developed an interesting method (named GWAS subtraction method) by comparing the lead loci identified by genomic SEM with those reaching genome-wide significance in single-input GWAS, which can effectively discover more valuable novel loci.

**Fine Mapping**

To identify the most plausible causal variants associated with PVD, we used SuSIE and FINEMAP [9], implemented in the R package echolocatoR v.2.0.3. We set a probability threshold of 0.95 to define credible sets of potentially causal variants. Here are the detailed steps:

Identifying Causal Variants with SuSIE and FINEMAP: SuSIE (Sum of Single Effects) and FINEMAP are tools for fine-mapping analysis aimed at determining the most likely causal variants associated with a phenotype. In this step, we used a 250 kb window to include regions associated with each lead SNP and calculated the posterior probability of causality for each SNP within these regions.

Credible Sets: We set a probability threshold of 0.95, and if a variant's posterior probability exceeded this threshold, it was considered a potential causal variant.

Consensus SNPs and Probability Sets: echolocatoR defines a 'consensus SNP' as a variant that appears in both SuSIE and FINEMAP results. For these consensus SNPs, the tool calculates their average posterior probability and determines an average credible set (set to 1 when the mean SNP-wise posterior probability across SuSIE and FINEMAP exceeds 0.95 and 0 otherwise).

**Transcriptome-Wide Association Study (TWAS)**

After identifying potential causal variants, we conducted a TWAS to prioritize genes associated with PVD based on the relationship between gene expression and phenotype. We used the FUSION method for TWAS, utilizing 37,920 precomputed eQTL features from GTEx v.8 (gene/tissue pairs) to calculate expression associations between different genes and tissues.

Further Analysis of TWAS Results: The PVD data contained sufficient variants to analyze 36,149 features (out of 37,920 eQTL features), indicating high data quality. Genes with P values less than 0.05 (significantly associated with the PVD structural equation) were included in further analysis.

FOCUS Method for Prioritizing PVD Genes: For these TWAS-significant genes, we further applied the FOCUS method (a fine-mapping method designed for TWAS studies) to prioritize PVD genes. FOCUS assesses the causal relationship between genes and phenotypes based on posterior inclusion probability. Following previous studies, we considered TWAS-significant genes that were consistent with other evidence (such as FOCUS) as potentially causal.

**Gene Set and Disease Ontology Enrichment Analysis**

We performed gene enrichment and pathway set analysis using MAGMA and FUMA (GESA) data to investigate the potential relationships of PVD with Mendelian disease genes and associated pathways.

**Cell-Type Annotation Analysis**

To identify etiological cell types associated with PVD, we integrated single-cell RNA sequencing data using CELLECT [10]. We used the Tabula Muris dataset, which contains transcriptomic data from 100,000 cells and 20 organs and tissues of Mus musculus [11]. We preprocessed and normalized the data using CELLEX, calculating expression specificity likelihood scores for each gene. We then performed cell-type-specific analysis using the ldsc software, classified cell types, and applied a false discovery rate (FDR) threshold of 0.05.

**Genetic Contribution of Genomic Regions**

The LDSC tool was used to calculate partitioned heritability, which allocates the genetic information of a phenotype to different genomic regions (such as genes, enhancers, and repressors) to assess their contributions to phenotypic heritability. Specifically, LDSC uses weighted LD matrices, genotype frequency files, and summary statistics for calculation, ultimately estimating the genetic contribution of each region.

**Cross-Phenotype Genetic Correlations**

To identify the associations between the constructed unmeasured PVD GWAS analysis data and previously studied diseases and biomarkers, we performed large-scale association analyses. We used the iCPAGdb database for cross-phenotype analysis to study the genetic correlations between PVD GWAS and other traits. We implemented Mendelian randomization (MR) analysis using the MendelianRandomization R package v.0.7.0, with inverse variance weighting (IVW) as the primary method for correlation assessment (*P < 0.05*).

**Polygenic Risk Score Construction Based on Summary Data**

We calculated polygenic risk scores (PRS) based on genome-wide summary statistics and assessed the genetic contribution of different chromosomal regions to the disease. Specifically, we used the PRS-CS (Polygenic Risk Score with Continuous Shrinkage) software to estimate the posterior effect sizes of SNPs using GWAS data and an external LD reference panel, thereby calculating the PRS. This method employs Bayesian regression models to estimate effect sizes based on GWAS summary statistics and integrates LD reference panels to compute PRS.
